# Organizing Pneumonia Associated with Pegylated Interferon *α* and Ribavirin Therapy

**DOI:** 10.1155/2015/794592

**Published:** 2015-01-29

**Authors:** Amit Chopra, Creticus Marak, Narendrakumar Alappan, Chang Shim

**Affiliations:** ^1^Division of Pulmonary and Critical Care, Albany Medical Center, Albany, NY, USA; ^2^Division of Pulmonary and Critical Care, National Jewish Health, Denver, CO, USA; ^3^Division of Pulmonary and Critical Care, Cleveland Clinic, Cleveland, OH, USA; ^4^Division of Pulmonary and Critical Care, Jacobi Medical Center, Albert Einstein College of Medicine, Bronx, NY, USA

## Abstract

Hepatitis C virus infection is the leading cause of chronic liver disease in the United States of America. Pegylated interferon *α* and ribavirin combination is the mainstay of treatment. Severe pulmonary toxicities are rarely reported. We report here a case of severe form of organizing pneumonia secondary to pegylated interferon *α* therapy presenting as acute respiratory failure. Patient has near complete recovery with withdrawal of pegylated interferon *α* and steroid therapy. We report this case to raise the awareness of this rare but potentially life-threatening pulmonary complication of pegylated interferon *α* therapy.

## 1. Introduction

Hepatitis C virus infection is a major public health problem worldwide. In the United States it is the leading cause of chronic liver disease, death from liver disease, and indication for liver transplantation [1]. According to Association for the Study of Liver Disease 2009 [2] guidelines, pegylated interferon *α* (2a or 2b) and ribavirin combination is the mainstay of treatment. Most common treatment related side effects reported are flu-like symptoms, gastrointestinal disturbances, psychiatric disorders, and hematological abnormalities such as anemia and leukopenia [3]. Severe pulmonary toxicities are rarely reported [4]. We report here a case of severe form of organizing pneumonia secondary to pegylated IFN-*α* therapy. A thorough MEDLINE search revealed only six case reports of organizing pneumonia (previously called BOOP) secondary to the IFN-*α* therapy (three from pegylated IFN-*α*) but none of the cases had acute respiratory failure and ARDS.

## 2. Case Report

A 65-year-old Hispanic male former smoker with past medical history significant for noninsulin dependent diabetes and chronic hepatitis C infection (genotype 1b) with cirrhosis was admitted to our hospital with nonproductive cough and progressively worsening shortness of breath of one month duration. He was a former intravenous drug user and was diagnosed with hepatitis C infection 20 years ago. His laboratory analysis showed persistently elevated transaminases level with aspartate aminotransferase AST and alanine aminotransferase ALT levels between 50 U/L–80 U/L and elevated HCV RNA copies. Liver biopsy showed chronic active hepatitis with minimal activity and mild cirrhosis. He was started on pegylated interferon *α*-2a 180 mcg/week subcutaneously and oral ribavirin 1000 g/day. He has good response on this therapy with normalization of the transaminases with undetectable HCV viral loads. He tolerated therapy with minor side effects of mild anemia and fatigue. After nine months of therapy he began to have nonproductive cough, low grade fever, and shortness of breath which progressively decreased to the point that he became extremely short of breath while performing routine activities such as changing clothes or walking to the bathroom. There was no history of sick contact, recent travel, or hazardous chemical or occupational exposures. On admission his blood pressure was 122/63 mm Hg, pulse rate 99/minute, respiratory rate 28/minute, oral temp 98 F, and oxygen saturation 88% on room air. He was noted to be in moderate respiratory distress with use of accessory muscle and was unable to speak in full sentences. Physical examination was remarkable for diffuse bilateral coarse inspiratory crackles; however, the other systems were within normal limits.

**Figure 1:**
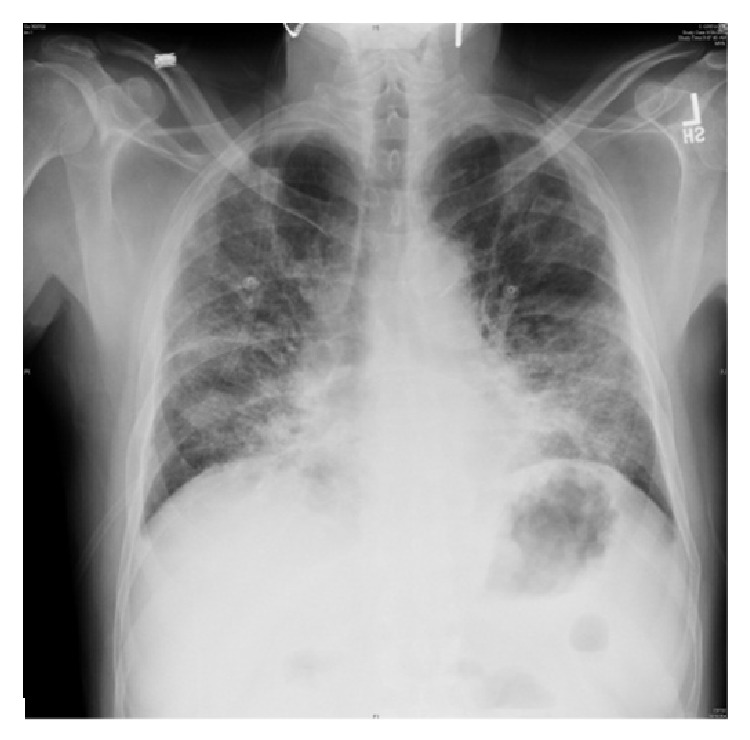
CXR, frontal view before treatment, showing bilateral interstitial opacities with small lung volume.

**Figure 2:**
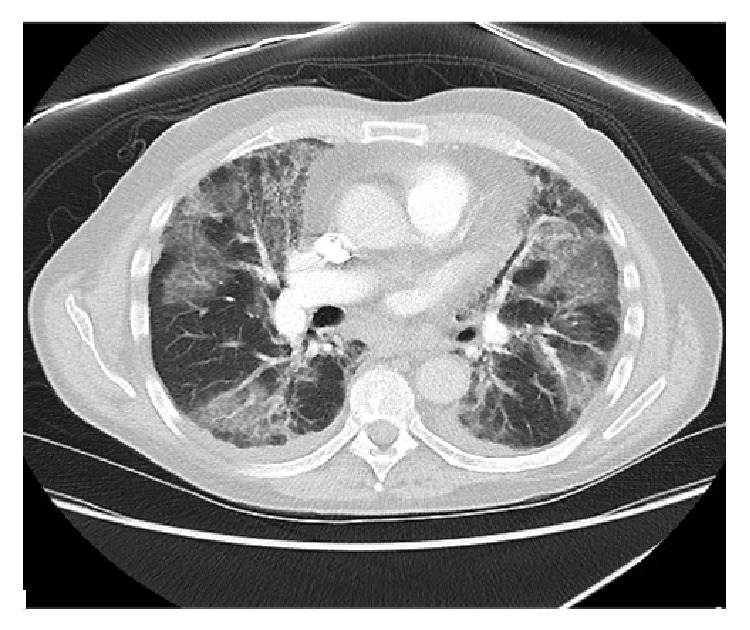
CT scan, representative cut showing b/l ground glass opacities with septal thickening from acute lung injury.

**Figure 3:**
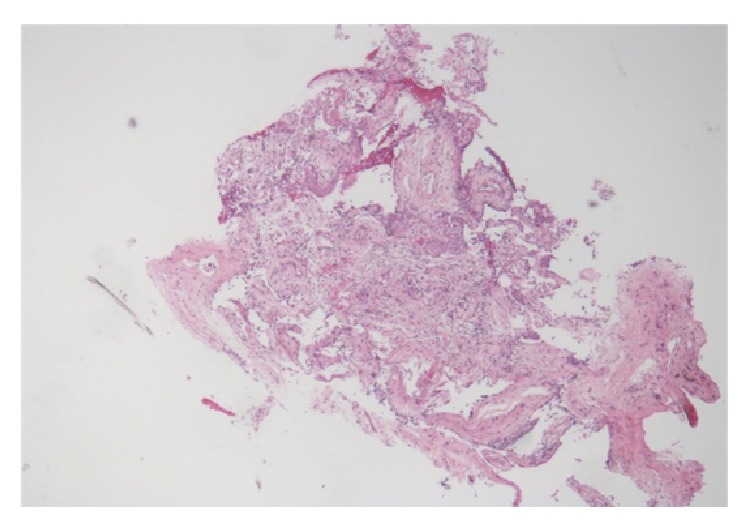
Histopathology, focal organizing pneumonia with chronic inflammation and focal fibrinous exudates.

**Figure 4:**
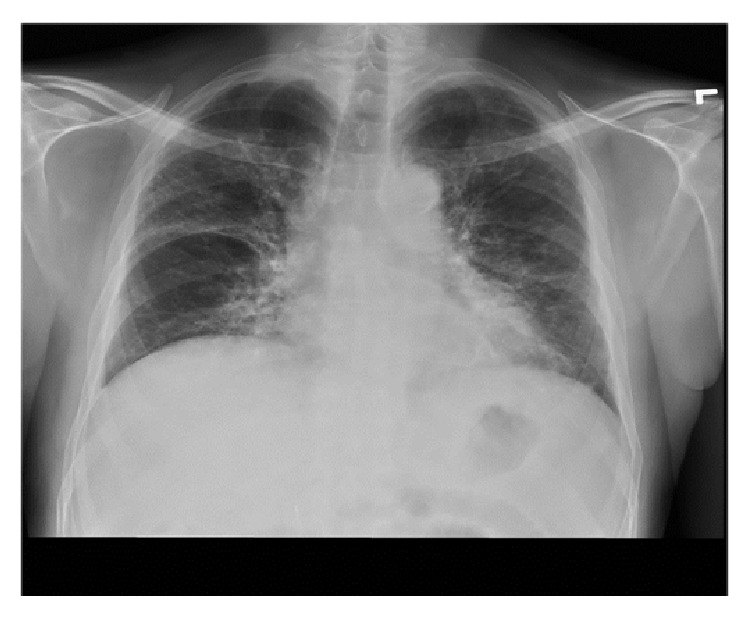
CXR, frontal view after 3 months of steroid therapy with marked improvement in interstitial opacities and residual fibrotic changes.

Laboratory investigation revealed WBC of 3.8 cell/*μ*L with normal differentials, hemoglobin of 10 gm/dL, platelet count of 110 k/*μ*L, AST of 33 U/L, and ALT 12 U/L. Initial chest radiograph (CXR) (Figure 1) revealed bilateral diffuse patchy opacities. CT chest (Figure 2) showed bilateral patchy areas of ground glass opacities in geographic distribution associated with septal thickening. He was empirically started on intravenous antibiotics Ceftriaxone 1000 mg/day and Azithromycin 500 mg/day for presumed community acquired pneumonia and he underwent flexible bronchoscopy the following day. Bronchoscopy revealed friable bronchial mucosa with minimal clear secretions but no other abnormality. Bronchoalveolar lavage (BAL) and multiple transbronchial biopsies were obtained from the right middle and lower lobes. The cultures for bacteria, fungal, viral, pneumocystis carinii pneumonia (PCP), and mycobacteria were negative in BAL fluid. Histopathology of the transbronchial biopsy revealed edematous bronchial wall with adjacent alveolar tissue showing focal organizing pneumonia with chronic inflammation and focal fibrinous exudates (Figure 3). Immunostains for cytomegalovirus (CMV) and herpes simplex virus (HSV) were negative. There was no evidence of granulomas, fungi, or acid fast organisms. Subsequently on day two of his admission he developed acute respiratory distress requiring intubation and mechanical ventilator support. His clinical presentation was compatible with ARDS given (1) CXR with diffuse bilateral infiltrate, (2) hypoxia with PaO_2_/FiO_2_ = 109 (<200), and (3) no evidence of left ventricular dysfunction on echocardiogram. Given no obvious sign or symptoms of infection with negative BAL, he was empirically started on intravenous methyl prednisone at 40 mg every six hours, which resulted in dramatic improvement in his clinical status enabling him to be liberated from the mechanical ventilator on the 10th day of admission. Intravenous methyl prednisone was later switched to oral prednisone, initially at a dose of 1 mg/Kg with slow steroid taper. He was discharged to home on oxygen via nasal cannula. At 6-month followup, his symptoms have significantly improved with a good exercise tolerance and his CXR (Figure 4) and lung functions have also improved.

## 3. Discussion

Interferons are naturally occurring cytokines produced in response to the various stimuli and play important role in host's defense against viral infection, parasitic infection, and tumors [9]. Interferon-*α* synthesized by recombinant DNA technology is used in combination with ribavirin for the treatment of chronic hepatitis C [2]. IFN-*α* has several effects such as antiviral activity, growth regulation, inhibition of angiogenesis, regulation of cell differentiation, enhancement of major histocompatibility complex antigen expression, and enhancement of the activity of natural killer cells and cytotoxic T lymphocytes [10]. IFN toxicity is generally dose and duration dependent. Commonly associated adverse effects include flu like symptoms, thrombocytopenia, leukopenia, anemia, depression, autoimmune thyroiditis, and seizures [2].

Pulmonary toxicity is rare but can be potentially fatal with reported incidence of 0.4% to less than 1% [4, 11]. The spectrum of pulmonary toxicity associated with the use of IFN-*α* is diverse with commonly reported pulmonary toxicities being interstitial pneumonitis and sarcoidosis-like lesions followed by few case reports of pleural effusion, exacerbation of asthma, and secondary organizing pneumonia [4, 12]. The precise mechanism of IFN-*α* related pulmonary toxicity is not clear. The proposed mechanisms for the activity and pulmonary toxicity associated with IFN include inhibition of suppressor T cells, enhancement of cytotoxic T cells, induction of proinflammatory cytokines, and exaggerated release of fibrinogenic cytokines, such as platelet-derived growth factor and transforming growth factor–b, leading to lung injury [13]. Typically, cell-mediated pneumonitis has a strong relationship with accumulated dosage and a high degree of reversibility. Pegylated interferon *α* has a longer absorption and elimination half-life resulting in higher blood levels because of attachment of a polyethylene glycol chain (pegylation) compared to the conventional IFN-*α*. However, this may be associated with more pulmonary toxicity from drug accumulation [10].

Organizing pneumonia (OP), historically called bronchiolitis obliterans organizing pneumonia (BOOP), is a rare inflammatory lung disease involving the distal bronchioles, respiratory bronchioles, bronchiolar ducts, and alveoli. It is characterized by the presence of granulation tissue that obstructs the small bronchioles and extends into the distal alveolar ducts and alveoli. OP can be cryptogenic (unknown cause) or secondary to nonspecific response to lung injury. According to ATS/ERS classification cryptogenic organizing pneumonia is categorized as a subtype of idiopathic interstitial pneumonia [14]. On thorough review of the literature in English language we found a total of six cases of organizing pneumonia that have been associated with IFN-*α*/ribavirin combination therapy for chronic hepatitis C infection (summarized in Table 1) [4–8]. Three of six cases were associated with pegylated IFN-*α*. They were mild to moderate cases of OP and resolved completely after withdrawal of IFN therapy with or without the treatment with steroids. Our patient presented with severe form of pulmonary toxicity resulting in diffuse lung involvement with acute respiratory distress syndrome. Lung imaging, histopathological findings, and response to steroids were compatible with the diagnosis of the organizing pneumonia. Although ribavirin can cause dry cough and dyspnea, there are no documented cases of another pathologic pulmonary toxicity due to ribavirin therapy alone. This patient had mild anemia and thrombocytopenia from IFN-*α* therapy and detailed clinical evaluation for other secondary causes of OP was negative. Altogether, his case strongly supports the diagnosis of organizing pneumonia secondary to IFN-*α* therapy.

With the institution of IFN-*α*/ribavirin therapy for chronic hepatitis C, the outcome has improved for this major public health problem. It is anticipated that the use of IFN for treatment of chronic hepatitis C will increase in the future. We report this case to raise the awareness of this rare but potentially life-threatening pulmonary complication of interferon *α* therapy.

## Figures and Tables

**Table 1:** Summary of published case reports in English language of organizing pneumonia associated with IFN-alfa and ribavirin in chronic hepatitis C.

Author (year)	Cases	Age/sex	Type of IFN	Combination with ribavirin	Duration of therapy (weeks)	Outcome	Genotype	Reference
Ogata et al. (1994)	1	64/m	IFN *α*-2b	No	11	Resolved with treatment	Unknown	[5]
Kumar et al. (2002)	2	50/f	IFN *α*-2b	Yes	24	Resolved without treatment	1b	[4]
		41/f	IFN *α*-2b	Yes	10	Resolved without treatment	Unknown	
Crespi et al. (2008)	1	63/m	PEG-IFN alfa-2a	Yes	8	Resolved with treatment	2a/2c	[6]
Vila et al. (2008)	1	49/m	PEG-IFN alfa-2b	Yes	24	Resolved with treatment	1b	[7]
Martins et al. (2012)	1	67/f	PEG-IFN alfa-2a	Yes	36	Resolved with treatment	1b	[8]
Current case	1	65/m	PEG-IFN alfa-2a	Yes	36	Improved with treatment	Unknown	
